# Defective early innate immune response to ectromelia virus in the draining lymph nodes of aged mice due to impaired dendritic cell accumulation

**DOI:** 10.1111/acel.13170

**Published:** 2020-07-12

**Authors:** Colby Stotesbury, Eric B. Wong, Lingjuan Tang, Brian Montoya, Cory J. Knudson, Carolina R. Melo‐Silva, Luis J. Sigal

**Affiliations:** ^1^ Department of Microbiology and Immunology Thomas Jefferson University Philadelphia PA USA

**Keywords:** chemokines, dendritic cells, ectromelia virus, innate immunity, monocytes, natural killer cells

## Abstract

It is known that aging decreases natural resistance to viral diseases due to dysfunctional innate and adaptive immune responses, but the nature of these dysfunctions, particularly in regard to innate immunity, is not well understood. We have previously shown that C57BL/6J (B6) mice lose their natural resistance to footpad infection with ectromelia virus (ECTV) due to impaired maturation and recruitment of natural killer (NK) cells to the draining popliteal lymph node (dLN). More recently, we have also shown that in young B6 mice infected with ECTV, the recruitment of NK cells is dependent on a complex cascade whereby migratory dendritic cells (mDCs) traffic from the skin to the dLN, where they produce CCL2 and CCL7 to recruit inflammatory monocytes (iMOs). In the dLN, mDCs also upregulate NKG2D ligands to induce interferon gamma (IFN‐γ) expression by group 1 innate lymphoid cells (G1‐ILCs), mostly NK in cells but also some ILC1. In response to the IFN‐γ, the incoming uninfected iMOs secret CXCL9 to recruit the critical NK cells. Here, we show that in aged B6 mice, the trafficking of mDCs to the dLN in response to ECTV is decreased, resulting in impaired IFN‐γ expression by G1‐ILCs, reduced accumulation of iMOs, and attenuated CXCL9 production by iMOs, which likely contributes to decrease in NK cell recruitment. Together, these data indicate that defects in the mDC response to viral infection during aging result in a reduced innate immune response in the dLN and contribute to increased susceptibility to viral disease in the aged.

## Graphical abstract

Stotesbury et al. show that in aged mice, the trafficking of migratory dendritic cells (mDC) to the draining lymph node (dLN) in response to viral infection is decreased. Resulting in impaired expression of IFN‐γ by Group 1 Innate Lymphoid cells, reduced recruitment of and attenuated CXCL9 production by inflammatory monocytes (iMO). As a consequence, natural killer cell recruitment to the dLN is increased and susceptibility to lethal viral disease is increased.

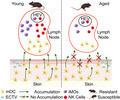

## 1 INTRODUCTION

It is known that aging negatively affects the ability of the host immune system to respond to viral infections (Nikolich‐Zugich, 2018). Research centered on identifying age‐related changes of the immune system has led to the discovery of expansive defects within the adaptive immune system, such as the generation and maintenance of lymphocytes, altered cytokine profiles, B‐ and T‐cell recall responses, and the acquisition of “age‐associated B cells” (Brien, Uhrlaub, Hirsch, Wiley, & Nikolich‐Zugich, 2009; Frasca, Diaz, Romero, & Blomberg, 2016; Hao, O'Neill, Naradikian, Scholz, & Cancro, 2011; Jiang, Fisher, & Murasko, 2011). However, studies examining age‐related changes of the innate immune system remain scarce.

Dendritic cells (DCs) are cells of the innate immune system which are well known for their role as professional antigen‐presenting cells for the priming of T‐cell responses and that are characterized by the expression of the integrin CD11c (Merad, Sathe, Helft, Miller, & Mortha, 2013). Migratory DCs (mDCs) are a class of DCs that have the ability to migrate from tissues to secondary lymphoid organs such as lymph nodes (LNs) (Worbs, Hammerschmidt, & Forster, 2017). mDCs are the predominant class of DCs in the skin. They constitutively migrate from the skin to the local LN and increase their migration during infection. Because they are mature, migrant mDCs can be distinguished from other DCs in LNs by high levels of expression of major histocompatibility (MHC) class II (MHC II) molecules at the cell surface (MHC II^hi^) (Miller et al., 2012). Skin mDCs are best known for their ability to present antigens to T‐cells in the dLN in various models of infection, immunization, and contact hypersensitivity (Bollampalli et al., 2015; Bouteau et al., 2019; Kaplan, Jenison, Saeland, Shlomchik, & Shlomchik, 2005). However, they can also play an important role at initiating the innate immune response cascade in the dLN (Wong et al., 2018).

It is known that the functionality of DCs, including mDCs, can decrease with age. For example in aged mice, defects in conventional DCs can negatively affect anti‐tumor NK and T‐cell responses (Grolleau‐Julius, Harning, Abernathy, & Yung, 2008; Guo, Tilburgs, Wong, & Strominger, 2014a, 2014b), and mDCs of the lung migrate poorly to the dLN during respiratory syncytial virus (RSV) infection resulting in decreased T‐cell responses (Zhao, Zhao, Legge, & Perlman, 2011). In humans, the numbers of various DC types are reduced with age (Della Bella et al., 2007; Gupta, 2014), and monocyte‐derived dendritic cells display poor antigen uptake (Agrawal et al., 2007). Moreover, in both mice and humans, aging results in reduced numbers of Langerhans cells (a type of mDC) in the skin, and impaired migration (Cumberbatch, Dearman, & Kimber, 2002; Pilkington et al., 2018).

Ectromelia virus (ECTV) is an *Orthopoxvirus* and a natural pathogen of the mouse which naturally enters the body through the skin, most frequently of the footpad. Footpad infection with ECTV causes a lethal disease known as mousepox in susceptible strains of mice such as BALB/c, but not in mousepox‐resistant mice, such as young C57BL/6 (B6) (Wallace, Buller, & Morse, 1985). In both, susceptible and resistant mouse strains, ECTV spreads lymphohematogenously from the footpad to the local popliteal draining LN (dLN) and then the blood, eventually infecting the liver and spleen (Esteban & Buller, 2005; Sigal, 2016). Resistant mice survive because compared to susceptible mice, they control better the systemic spread of the virus from the dLN and also viral replication in spleen and liver.

While the dLN is largely thought of as the site where T‐cell priming occurs (Hickman et al., 2008), it also serves as a site where innate immune mechanisms prevent lymphohematogenous viral dissemination. In a series of papers, we have previously shown an intricate network of collaborative innate immune responses within the dLN that lead to the control and, ultimately, resolution of ECTV infection in young B6 mice (Fang, Roscoe, & Sigal, 2010; Wong et al., 2018; Xu et al., 2015). Within this inflammatory network, skin mDCs (CD11c^+^ MHC II^hi^), play a central organizing role. Specifically, we showed that soon after ECTV infection in the footpad, CD11c^+^ MHC II^hi^ mDCs increase their migration from the skin of the footpad to the dLN. Once in the dLN, infected and uninfected mDCs produce a variety of inflammatory mediators. Among these, the chemokines CCL2 and CCL7 recruit inflammatory monocytes from the blood into the dLN (Wong et al., 2018). In the dLN, infected mDCs also upregulate ligands for NKG2D, such as MULT1, to predominantly induce the production of interferon gamma (IFN‐γ) in NK cells and in some of the few innate lymphoid cells 1 (ILC1) already present in the dLN (Wong et al., 2018). Together, NK cells and ILC1 constitute the Group‐1 Innate Lymphoid Cells (G1‐ILCs), which are characterized by their ability to produce IFN‐γ and their expression of NK1.1 and NKp46. The IFN‐γ produced by G1‐ILCs activates the uninfected newly arrived iMOs which, in response, produce the chemokine CXCL9 to recruit circulating mature NK cells into the dLN (Wong et al., 2018). These incoming NK cells have a critical role at curbing systemic virus spread from the dLN (Fang et al., 2010). Of note, once they get infected, iMOs do not produce CXCL9 but become the major producers of Type I interferon (IFN‐I), which is also critical for the control of virus spread and resistance to mousepox (Jacoby, Bhatt, & Brownstein, 1989; Karupiah, Fredrickson, Holmes, Khairallah, & Buller, 1993; Xu et al., 2015). Induction of IFN‐I in iMOs requires their infection. Notably, disruption of mDC migration to the dLN impairs the recruitment of NK cells and iMOs and results in susceptibility to mousepox (Wong, Montoya, Stotesbury, et al., 2019; Wong et al., 2018).

B6 mice older than 16 months fail to recruit NK cells to the dLN and are highly susceptible to mousepox (Fang et al., 2010; Fenner, 1949; Wallace et al., 1985), mimicking the increased susceptibility to viral infections observed in the elderly. The deficient NK cell migration to the dLN in aged mice is partly intrinsic, as their numbers are decreased in the circulation and have an immature phenotype in multiple tissues when compared to NK cells in young mice (Beli et al., 2011; Nair, Fang, & Sigal, 2015; Shehata, Hoebe, & Chougnet, 2015). Yet, adoptive transfer of NK cells from young mice only partially restores resistance to mousepox in aged B6 mice (Fang et al., 2010), suggesting other mechanisms contribute to the loss of resistance. Given the critical role of mDCs, G1‐ILC, and iMOs in resistance to mousepox (Wong, Montoya, Stotesbury, et al., 2019; Wong et al., 2018), we sought to investigate their role in age‐related susceptibility to viral disease.

## 2 RESULTS

### 2.1 Migratory dendritic cells are equally present in the footpad of naïve young and aged mice, but fail to accumulate in the dLN of aged mice during ECTV infection

**FIGURE 1:**
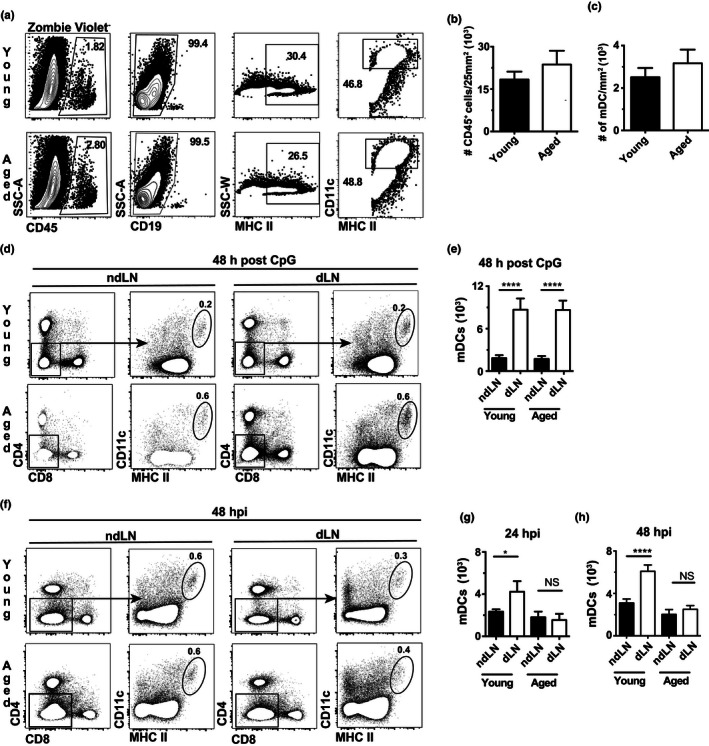
Migratory dendritic cells are present in the naïve footpad of aged mice at similar frequency, but fail to accumulate in the dLN during ECTV infection. mDCs in the footpad of naïve young and aged mice were analyzed. (a) Representative flow cytometry plots indicating the gating strategy. (b) Number of CD45^+^ cells/25 mm^2^ displayed as mean ± *SEM* of individual mice. (c) Number of mDCs (CD19^‐^ MHCII^+^ CD11c^hi^)/25 mm^2^ displayed as mean ± *SEM* of individual mice. Data correspond to two independent experiments combined with a total of 10–15 mice/group. (d) Representative flow cytometry plots showing the gating of mDCs (CD4^‐^ CD8^‐^ CD11c^+^ MHCII^hi^) in the ndLN and dLN of mice young and aged mice at 48 postinoculation with 50 µg CpG. (e) Total number of mDCs as mean ± *SEM* in the ndLN and dLN of individual aged and young mice at 48 hr postinoculation with 50 µg CpG in the footpad. Data correspond to three independent experiments combined with a total of 6–9 mice/group. (f) Representative flow cytometry plots for the gating of mDCs (CD4^‐^ CD8^‐^ CD11c^+^ MHCII^hi^) in the ndLN and dLN of young and aged mice at 48 hpi with 3,000 pfu ECTV‐dsRed in the footpad. (g) Total number of mDCs as mean ± *SEM* in the ndLN and dLN of individual aged and young mice at 24 hpi. Data correspond to two independent experiments combined with a total of 6–7 mice/group. (h) As in G but at 48 hpi. Data correspond to five independent experiments combined with a total of 25–26 mice/group. For all, *p < .05; ****p < .0001

Using mice transgenic for photoactivatable green fluorescent protein, we have recently demonstrated that following footpad infection with ECTV, CD11c^+^ MHC II^hi^ mDCs migrate from the footpad to the dLN to coordinate the recruitment of iMOs and NK cells to the dLN (Wong, Montoya, Stotesbury, et al., 2019; Wong et al., 2018; Xu et al., 2015). To continue elucidating the mechanisms underlying age‐related susceptibility to mousepox, we used flow cytometry (gating strategy shown in (Figure 1a) to examine differences in mDCs within the skin of the footpad in naïve young and aged B6 mice. Results showed that young and aged mice had similar numbers of hematopoietic (CD45^+^) cells (Figure 1b) and mDCs, which in the skin are identified as (CD19^‐^ CD11c^hi^ MHC II^+^ (Figure 1c).

We next focused our attention onto the dLN. It is known that mDCs constitutively migrate in low numbers from the skin to the local LNs, and increase their migration after an inflammatory stimulus (Stoitzner, Tripp, Douillard, Saeland, & Romani, 2005; Wong, Montoya, Stotesbury, et al., 2019). To test whether aging may affect mDC accumulation in the dLN after a non‐infectious inflammatory stimulus, we inoculated young and aged mice in the left footpad with the TLR9 agonist CpG and compared the number of mDCs in the left popliteal dLN and the contralateral (right) popliteal non‐draining LN (ndLN) at 48 hr postinoculation. While aged mice had an increase in the frequency of mDCs in the dLNs and ndLNs (gating strategy, Figure 1d), this was due to a decrease in other LN populations, because both, aged and mice, had similar absolute numbers of mDCs in their ndLNs, and a marked and similar increase in the number of mDCs in their dLNs (Figure 1e). This suggests that the constitutive and TLR9‐ligand‐induced migration of mDCs to LNs is intact in aged mice. Next, we compared numbers of mDCs in dLNs and ndLNs in response to infection in the left footpad with ECTV expressing the red fluorescent protein dsRed (ECTV‐dsRed, an ECTV recombinant that is as virulent as WT ECTV (Roscoe, Xu, & Sigal, 2012). As above, aged mice had increased frequencies but similar absolute numbers of mDCs in their ndLNs (right popliteal LN) (gating strategy, Figure 1f–h) confirming similar constitutive migration. However, while as expected (Wong, Montoya, Stotesbury, et al., 2019), the number of mDCs increased significantly in the dLNs of young mice at 24 hpi (Figure 1g) and 48 hpi (Figure 1h), these increases were not observed in aged mice, suggesting early defective migration of mDCs to the dLN of aged mice in response to virulent ECTV infection.

### 2.2 Impaired early production of IFN‐γ by Group 1 ILCs in response to ECTV infection in aged mice

**FIGURE 2:**
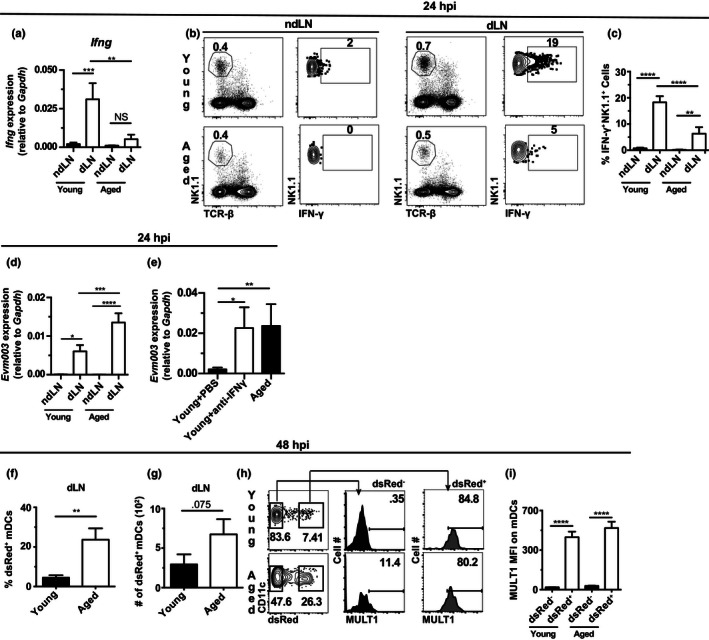
Impaired early production of IFN‐γ by Group 1 ILCs in response to ECTV infection in aged mice. (a) Expression of *Ifng* in the ndLN and dLN of young and aged mice determined by RT‐qPCR at 24 hpi with 3,000 pfu WT ECTV. Data shown as mean ± *SEM* of individual mice and correspond to four independent experiments combined with a total of 13–18 mice/group. (b) Representative flow cytometry plots of IFN‐γ production by G1‐ILCs (NK1.1^+^ TCRβ^‐^) at 24 hpi with 3,000 pfu of WT ECTV in the ndLN and dLN of aged and young mice. (c) Frequencies of IFN‐γ^+^ G1‐ILCs in the ndLN and dLN of young and aged mice determined by flow cytometry at 24 hpi with 3,000 pfu WT ECTV. Data correspond to the mean ± *SEM* of individual mice and to four independent experiments combined with a total of 14–21 mice/group. (d) Expression of the viral gene *Evm003* determined by RT‐qPCR in the ndLN and dLN of aged and young mice at 24 hpi with 3,000 pfu WT ECTV. Data shown as mean ± *SEM* correspond to four independent experiments combined with a total of 13–18 mice/group (e) Aged and young mice were injected intraperitoneally with 100 μg mouse IFN‐γ blocking monoclonal antibody 24 hr prior to infection with 3,000 pfu WT ECTV. Data show mean ± *SEM* relative expression of viral gene *Evm003* in the ndLN and dLN of individual mice determined by RT‐qPCR at 24 hpi. Data correspond to three independent experiments combined with a total of 10–13 mice/group. (f) Representative flow cytometry plots of MULT1 expression by ECTV‐dsRed^‐^ or ECTV‐dsRed^+^ mDCs (CD11c^+^ MHC II^hi^) in the dLN of aged and young mice at 48 hpi with 3,000 pfu ECTV‐dsRed. (g) MFI of MULT1 on ECTV‐dsRed^‐^ or ECTV‐dsRed^+^ on total mDCs (CD11c^+^ MHC II^hi^). MFI is shown as mean ± *SEM*. Data correspond to three independent experiments combined with a total of 13–14 mice/group. For all, *p < .05; **p < .0; ***p < .001; ****p < .0001

Having found defective accumulation of mDCs in the dLN of aged mice in response to WT ECTV infection, we investigated whether downstream innate immune mechanisms in the dLN were also disrupted. quantitative PCR of reverse‐transcribed RNA (RT‐qPCR) of whole dLNs at 24 hpi with WT ECTV showed that *Ifng* mRNA was lower in the dLN of aged than young mice (Figure 2a). Moreover, by flow cytometry, fewer G1‐ILCs (NK1.1^+^ TCRβ^‐^), most of which are NK cells (Wong et al., 2018), produced IFN‐γ in the dLN of aged compared to young mice (Figure 2b–c). These data demonstrate very early defects in the activation of dLN G1‐ILCs in aged mice. Of note, while IFN‐γ produced by G1‐ILCs is important for the activation of iMOs to recruit NK cells to the dLN (Wong et al., 2018), IFN‐γ can also directly inhibit viral replication in infected cells (Boehm, Klamp, Groot, & Howard, 1997). Consistent with this, viral transcripts in the dLN of aged mice at 24 hpi were increased (Figure 2d). This was likely due to the lower levels of IFN‐γ, because the dLN of young mice treated with IFN‐γ blocking antibody 24 hr before infection had levels of viral transcripts at 24 hpi that were similar to those in aged mice and significantly higher than in PBS‐treated control young mice (Figure 2e).

We have previously shown that infected mDCs in the dLN of young mice upregulate the NKG2D ligands Rae1 and MULT1 to induce IFN‐γ in G1‐ILCs through NKG2D. This was detected as *Rae1* transcripts in sorted total mDCs at 24 hpi, when the number of infected mDCs in the dLN is still too low for reliable flow cytometry analysis; as *Rae1* transcripts at 48 hpi in sorted infected (GFP^+^) mDCs in mice infected with ECTV expressing green fluorescent protein (ECTV‐GFP); and as surface expression of MULT1 protein at 48 hpi in infected mDCs (DsRed^+^) in mice infected with ECTV‐DsRed (Wong et al., 2018). Consistent with those results, we were unable to detect dsRed^+^ mDCs in the dLN of young or aged mice at 24 hpi with ECTV‐dsRed (not shown) so we were not able to determine NKG2D ligand expression at this time. However, at 48 hpi and despite the lower number of mDCs (Figure 1h), aged mice had significantly higher frequency and similar absolute numbers of dsRed^+^ mDCs in the dLN when compared to young mice (Figure 2f–g). This allowed us to determine MULT1 expression by flow cytometry. Notably, we found that mDCs upregulated MULT1 to similar levels in aged and young mice (Figure 2h–i) indicating that the infected mDCs of aged mice do not have defective NKG2D ligand upregulation. These data strongly suggest that the lack of IFN‐γ production by G1‐ILCs at 24 hpi in the dLNs of aged mice is due to the absence of mDC migration to the dLN rather than an intrinsic inability to stimulate G1‐ILCs through NKG2D ligand upregulation. Yet, intrinsic defects in G1‐ILCs cannot be excluded as a possible cause of IFN‐γ deficiency.

### 2.3 Decreased recruitment of iMOs to the dLN of aged mice in response to ECTV infection

**FIGURE 3:**
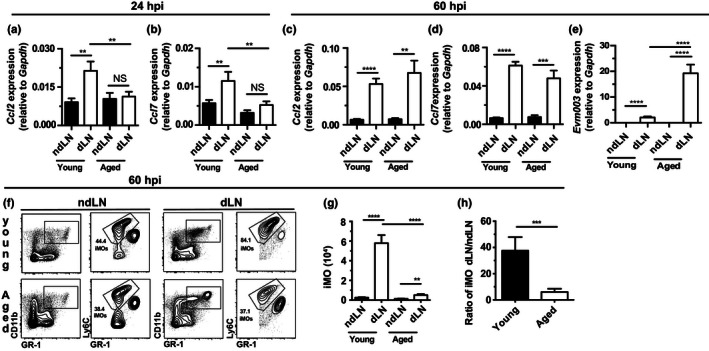
Decreased recruitment of iMOs to the dLN of aged mice in response to ECTV infection. (a–b) Expression of *Ccl2* (a) and *Ccl7* (b) in the ndLN and dLN of aged and young mice determined by RT‐qPCR at 24 hpi with 3,000 pfu WT ECTV. Data shown as mean ± *SEM* of individual mice. Data correspond to four independent experiments combined with a total of 13–18 mice/group. (c–d) Expression of *Ccl2* (c) and *Ccl7* (d) in the ndLN and dLN of aged and young mice determined by RT‐qPCR at 60 hpi with 3,000 pfu WT ECTV. Data shown as mean ± *SEM* of individual mice. Data correspond to three independent experiments combined with a total of 9–15 mice/group. (e) Expression of *Evm003* in the ndLN and dLN of aged and young mice determined by RT‐qPCR at 60 hpi with 3,000 pfu WT ECTV. Data shown as mean ± *SEM* of individual mice. Data correspond to five individual experiments combined with a total of 15–20 mice/group. (f) Concatenated flow cytometry plots from aged (*n* = 3) and young mice (*n* = 4) depicting the presence of iMOs (CD11b^+^ GR‐1^+^ Ly6C^+^) in the ndLN and dLNs of aged and young mice at 60 hpi with 3,000 pfu of ECTV‐GFP. (g‐h) Total numbers of iMOs in the ndLN and dLN (e) and dLN/ndLN iMOs ratio (f) in young and aged mice at 60 hpi with 3,000 pfu WT ECTV. Data shown as mean ± *SEM* of individual mice. Data correspond to four independent experiments combined with a total of 14–19 mice/group. For all, **p < .0; ***p < .001; ****p < .0001

We have shown that mDCs produce CCL2 and CCL7 to recruit iMO to the dLN (Wong et al., 2018; Wong, Montoya, Stotesbury, et al., 2019). Because aged mice had reduced numbers of mDCs in the dLN, we measured the expression of mRNA for these chemokines by RT‐qPCR. Compared to the ndLN, *Ccl2* and *Ccl7* mRNAs were significantly increased in the dLN of young but not aged mice at 24 hpi (Figure 3a,b). However, at 60 hpi, which is the peak of iMOs recruitment to the dLN in young mice (Xu et al., 2015), the relative quantities of *Ccl2* and *Ccl7* transcripts in the dLN of young and aged mice were similar (Figure 3c–d) while the virus loads, measured as viral transcripts, were ~10 fold higher in aged mice (Figure 3e). Yet, aged mice recruited significantly fewer iMOs to the dLN than young mice (Figure 3f–g), resulting in ~five‐fold reduction in the dLN/ndLN iMO ratio (Figure 3h). Thus, while expression levels of *Ccl2* and *Ccl7* at 60 hpi were similar, the absence of *Ccl2* and *Ccl7* at 24 hpi and decreased iMO recruitment at 60 hpi in the dLNs of aged mice suggest that aged mice suffer a delayed rather than absent innate immune response in the dLN, and/or that aged mice require much higher virus loads than young ones to fully activate their innate immune response.

### 2.4 Decreased production of CXCL9 by iMOs in the dLN of aged mice in response to ECTV

**FIGURE 4:**
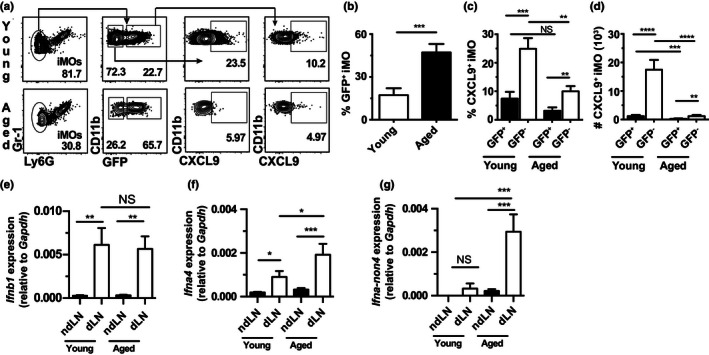
Decreased production of CXCL9 by iMOs in the dLN of aged mice in response to ECTV. (a) Representative flow cytometry plots of CXCL9 expression infected (GFP^+^) and uninfected (GFP^‐^) iMOs (CD11b^+^ GR‐1^+^ Ly6G^‐^) at 60 hpi with 3,000 pfu ECTV‐GFP in the dLN of the indicated mice. (b) Frequency of infected iMOs in the dLN of the indicated mice at 60 hpi with ECTV. Data shown as mean ± *SEM* of individual mice. Data correspond to three independent experiments combined with a total of 12–14 mice/group (c–d) Frequencies (c) and absolute numbers (d) of CXCL9^+^ infected and uninfected iMOs in the dLN of indicated mice at 60 hpi with ECTV. Data shown as mean ± *SEM* of individual mice. Data correspond to four individual experiments combined with a total of 19–23 mice/group. (e‐g) Expression of *Ifna4* (e)*, Ifna‐ non4* (f)*, and Ifnb* (g) in the ndLN and dLN of aged and young mice determined by RT‐qPCR at 60 hpi with 3,000 pfu WT ECTV. Data shown as mean ± *SEM* of individual mice. Data correspond to five individual experiments combined with a total of 16–20 mice/group

Upon recruitment to the dLN, iMOs become functionally divergent: in response to the IFN‐γ produced by G1‐ILCs, uninfected iMOs produce CXCL9 to recruit NK cells, while infected iMOs upregulate IFN‐I (Wong et al., 2018; Xu et al., 2015). Because IFN‐γ and iMOs were reduced in the dLN of aged mice, we investigated whether CXCL9 production by uninfected iMOs was impaired. To distinguish infected and uninfected iMOs, we infected mice with ECTV‐GFP and analyzed their dLNs by flow cytometry at 60 hpi (gating strategy depicted in Figure 4a). Compared to young mice, a significantly lower proportion of iMOs remained uninfected, the frequency of uninfected iMOs that produced CXCL9 was significantly reduced, and fewer uninfected iMOs produced CXCL9 in the dLN of aged mice (Figure 4b–d). Expression of the interferon type I (IFN‐I) gene *Ifnb1* was similar while *Ifna4* and *Ifna non4* (transcripts for all IFN‐α genes except IFN‐α4) were increased in the dLN of aged mice (Figure 4e–g). This increase in the expression of IFN‐α transcripts was likely due to the increased virus loads in the dLN of aged mice.

### 2.5 Defective anti‐viral innate immune responses in the dLN of aged mice are independent of viral virulence and lethality

**FIGURE 5:**
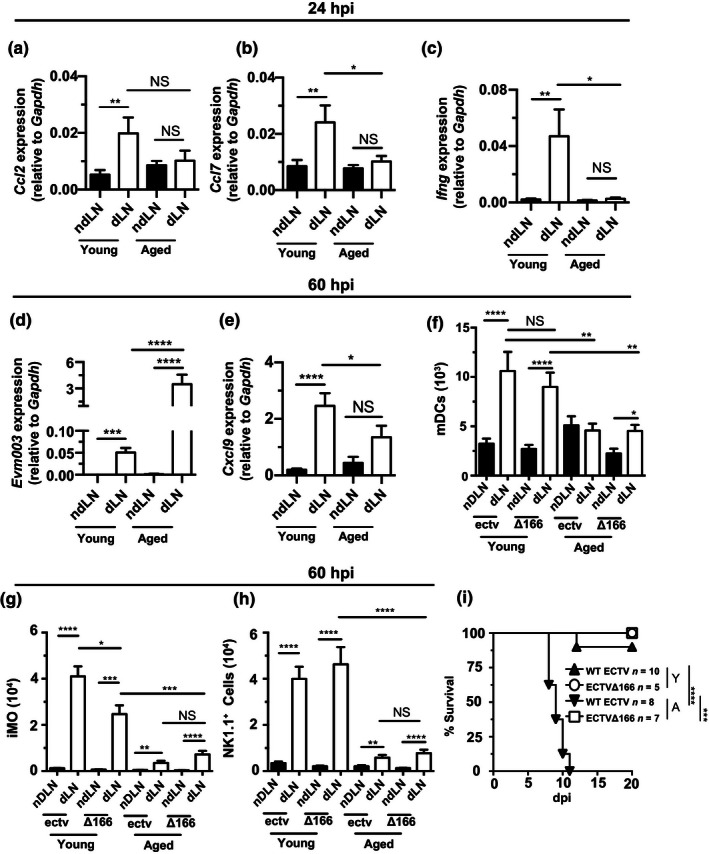
Defective anti‐viral innate immune responses in the dLN of aged mice are independent of viral virulence and lethality. (a–c) Expression of mRNA for *CCl2* (a), *Ccl7* (b), and *Ifng* (c) in the dLN of aged and young mice as determined by RT‐qPCR in individual mice at 24 hpi with 3,000 pfu ECTV‐Δ166. Data shown as mean ± *SEM* of individual mice. Data correspond to two independent experiments combined with a total of 6–9 mice/group. (d) Relative expression of viral gene *Evm003* was determined by RT‐qPCR in the ndLN and dLN of young and aged mice at 60 hpi with 3,000 pfu ECTV Δ166. Data are shown as mean ± *SEM* of individual mice and correspond to three independent experiments combined with a total of 6–9 mice/group. (e) Expression of *Cxcl9* mRNA was determined by RT‐qPCR in the ndLN and dLN of aged and young mice at 60 hpi with 3,000 pfu ECTV‐Δ166. Data shown as mean ± *SEM* of individual mice. Data correspond to two independent experiments combined with a total of 6–9 mice/group. (f) Total mDCs were determined in the ndLNs and dLNs of the indicated mice at 60 hpi with 3,000 pfu of ECTV‐Δ166. Data shown as mean ± *SEM* of individual mice. Data correspond to four independent experiments combined with a total of 6–15 mice/group. (g) Total numbers of iMOs (CD11b^+^ GR‐1^+^ Ly6C^+^) in the ndLN and dLN of aged and young mice at 60 hpi with 3,000 pfu ECTV‐GFP or ECTV‐Δ166. Data shown as mean ± *SEM* of individual mice. Data correspond to four independent experiments combined with a total of 8–18 mice/group. (h) Total number of NK cells in the ndLN and dLN of aged and young mice at 60 hpi with 3,000 pfu ECTV‐GFP or ECTV‐Δ166. Data shown as mean ± *SEM* of individual mice. Data correspond to four independent experiments combined with a total of 8–18 mice/group (i) The indicated mice were infected with 3,000 pfu ECTV‐GFP or ECTV Δ166 in the footpad. Survival was monitored. Data are displayed as a combination of two independent experiments with a total of 5–10 mice/group. For all, *p < .05; **p < .0; ***p < .001; ****p < .0001

To test whether the dysfunctional anti‐viral response in the dLN of aged mice was due to ECTV virulence or a general disability of aged innate immunity to rapidly respond to viral infection, we infected mice with ECTVΔ166, a mutant ECTV that lacks an IFN‐I decoy receptor and is at least 10^7^‐fold less virulent than WT ECTV (Xu et al., 2008). When compared to the dLN of young mice, the dLN of aged mice infected with ECTVΔ166 had decreased upregulation of *Ccl2, Ccl7,* and *Ifng* at 24 hpi (Figure 5a–c), and at 60 hpi, increased viral transcripts, decreased upregulation of *Cxcl9*, and reduced accumulation of mDCs, iMOs, and NK1.1^+^ cells (Figure 5d–h). Yet, while aged mice succumbed to WT ECTV, they survived ECTVΔ166 (Figure 5i) without signs of disease, indicating that a fast innate immune response in the dLN is important for resistance to virulent but not to highly attenuated ECTV.

## 3 DISCUSSION

Our previous work with ECTV led to the identification of molecular and cellular mechanisms responsible for the control of lymphohematogenous viral spread and corresponding protective immune responses. Briefly, we have previously identified an indispensable innate immune cascade against ECTV, whereby mDCs migrate to the dLN as early as 24 hpi, to recruit iMOs *via* CCL2/CCL7, and upregulate NKG2D ligands to induce IFN‐γ expression in G1‐ILCs (mostly NK cells present in the LN at steady state). Newly arrived uninfected iMOs sense this IFN‐γ and produce CXCL9 to recruit NK cells, while those iMOs that get infected iMOs lose CXCL9 expression but become major producers of IFN‐I (Wong et al., 2018; Wong, Montoya, Stotesbury, et al., 2019; Xu et al., 2015). In this study, we demonstrate that aged mice exhibit multiple defects within this immune cascade most likely triggered by defective mDC migration. We found that in young and aged mice, mDCs were in similar numbers in the skin and at steady state in LNs as determined in the ndLN of mice inoculated with CpG or infected with ECTV, and accumulated similarly in the dLN of mice that received CpG. However, upon ECTV infection, the accumulation of mDCs in the dLN of aged mice at 24 and 48 hpi was impaired. While our experiments do not directly analyze migration, our data suggest that the defective accumulation of mDCs in the dLN of aged mice is due to defective migration, because we have shown that following ECTV infection, the kinetics of migration of mDCs from the footpad to the dLN in young mice closely matches the kinetics of their accumulation (Wong, Montoya, Stotesbury, et al., 2019). Yet, while we have not found major differences in cell death using a viability marker (not shown) it still remains possible that viral infection causes increased death of mDCs in aged mice, and this could be partly responsible for the reduced accumulation of mDCs in the dLN that we observed.

Previous studies have shown that aged macrophages and DCs have an impaired response to TLR stimulus (Renshaw et al., 2002; Stout‐Delgado, Yang, Walker, Tesar, & Goldstein, 2008). It is known that TLR9/MyD88 signaling is required for resistance against ECTV (Rubio et al., 2013; Sutherland et al., 2011). Moreover, we have shown that TLR9/MyD88 in mDCs is required to recruit iMOs to the dLN (Xu et al., 2015). Here we demonstrate that the accumulation of mDCs in the dLN induced by a non‐infectious TLR9 agonist remains intact in aged mice. Yet, infection with WT ECTV or ECTV lacking a critical IFN‐I decoy receptor failed to induce mDC accumulation in the dLN of aged mice, suggesting that unknown changes in the skin as a result of aging, perhaps combined with ECTV immune evasion strategies other than IFN‐I, prevent the proper migration of mDC in response to infection in aged but not in young mice. ‬‬‬‬‬‬‬‬‬‬‬‬‬‬‬‬‬‬‬‬‬‬‬‬‬‬‬‬‬‬‬‬‬‬In this regard, in addition to the IFN‐I decoy receptor, ECTV encodes several cytokine and chemokine decoys (Sigal, 2016). It is possible that the skin of aged mice produces smaller amounts of a cytokine that is targeted by the virus, which is necessary for mDC migration. In addition, ECTV encodes a MyD88 inhibitor, and its effects could be greater in aged than in young mice. Future research should look into these issues.

It has been shown that conventional aged DCs fail to activate NK cells in a tumor model, leading to poor killing of tumor cells (Guo, Tilburgs, Wong, & Strominger, 2014a, 2014b). We have previously shown that following ECTV infection of young mice, a small but significant number of mDCs accumulate in the dLN of young mice and upregulate the transcription of NKG2D ligands. We have also demonstrated that at 48 hpi, the number of mDCs in the dLN is substantially increased and that infected but not uninfected mDCs upregulate the NKG2D ligand MULT1 at the cell surface (Wong, Montoya, Stotesbury, et al., 2019; Wong et al., 2018). Here we show that the increase in the number of mDCs in the dLN of aged mice does not occur either at 24 or 48 hpi. However, compared to young mice, a larger frequency of the mDCs in the dLN of age mice are infected and upregulate MULT1 infection resulting in roughly similar numbers of MULT1^+^ cells. The lack of accumulation of mDCs in the dLN of aged mice at 24 hpi can explain the significant reduction of CCL2/7 expression in the dLN at this time with a consequent reduction of iMOs recruitment at 60 hpi. Similarly, deficient accumulation of mDCs at 24 hpi and the resulting unavailability of mDCs expressing NKG2D ligands in the dLN can also explain the absence of IFN‐γ expression by G1‐ILCs at 24 hpi and the consequent decrease of CXCL9 expression by uninfected iMOs. Yet, an intrinsic defect in G1‐ILCs cannot be disregarded as we and others have shown intrinsic NK cell defects in aged mice (Beli et al., 2014; Fang et al., 2010; Nair et al., 2015). Notably, the decrease in mDC accumulation, IFN‐γ production, iMOs recruitment, CXCL9 expression, and NK cell migration was independent of viral pathogenicity, as infection with attenuated ECTV failed to restore these processes. Interestingly, even in the absence of this innate immune cascade, aged mice controlled attenuated ECTV.

iMOs are recruited to the dLN by CCL2 and CCL7 produced by infected mDCs. Incoming uninfected iMOs produce CXCL9 in response to IFN‐γ to recruit NK cells to the dLN while infected iMOs produce anti‐viral IFN‐I but not CXCL9 (Wong et al., 2018; Wong, Montoya, Stotesbury, et al., 2019). Here we show that aged mice recruit considerably fewer iMOs to the dLN. It is known that the frequency and numbers of circulating monocytes are unchanged in aged humans but increased in aged mice (Puchta et al., 2016). Therefore, the decrease in iMO recruitment to the dLN is most likely due to reduced CCL2/7 chemotactic signals. CXCL9 production by uninfected iMOs was significantly reduced which was most likely the result of impaired production of IFN‐γ by G1‐ILCs and increased frequency of infected iMOs. On the other hand, IFN‐I production appears to be intact in aged mice. Indeed, the overall expression of IFN‐I in the dLN of aged mice was higher than in young mice, which could be due to the increased frequency of infected iMOs, and the increase in viral loads due to deficient production of IFN‐γ by G1‐ILC.

As we have shown previously, NK cells fail to migrate to the dLN during ECTVΔ166 infection (Fang et al., 2010). Here we show that also mDCs fail to accumulate in the dLN during ECTVΔ166 infection which, similar to a virulent ECTV infection, results in absent *Ifng* upregulation and, likely as a consequence, reduced number of CXCL9‐producing iMOs. Notably aged mice infected with ECTVΔ166 survived despite this disruption of the innate immune cascade. Resistance to ECTVΔ166 challenge was most likely due to unabated IFN‐I production and sensing, culminating in the induction of a virus‐specific CD8 T‐cell response (Fang et al., 2010).

Our data suggest a delay in the first 24 hr of the immune response against ECTV. An interesting avenue to pursue would be to determine whether treating aged mice with CpG or IFN‐γ prior to or during active infection could promote survival to virulent ECTV. However, the mechanisms by which these treatments could protect aged mice is unpredictable, and its elucidation would require substantive work. For example, IFN‐γ supplementation could restore iMO activation, but could also rapidly decrease viral replication and preclude or diminish the innate responses in the dLN. Similarly, by curbing viral replication in the footpad, administration of CpG prior to ECTV infection may protect from lethality but, by drastically reducing virus loads, could result in decreased responses to ECTV in the dLN as we have previously observed in cGAS deficient mice treated with the STING ligand cGAMP (Wong, Montoya, Ferez, Stotesbury, & Sigal, 2019). Thus, while pathogen‐associated molecular patterns have immense potential as anti‐viral treatments for the aged, extensive studies would be required to determine their mechanism of action.

In summary, aging impairs mDC accumulation in the dLN during virulent or attenuated viral infection, leading to decreased IFN‐γ production by G1‐ILCs and impaired CXCL9 expression by uninfected iMOs, culminating in reduced NK cell recruitment to the dLN. Notably, the IFN‐I response of aged mice appears to compensate for ablated NK cell recruitment because aged mice survived ECTV Δ166 infection. Whether similar innate immune deficiencies of the age also apply to other tissues, such as DC, Imo, and NK cell migration to and function in the lung during SARS‐CoV‐2 infection, should be explored as a possible explanation for the increased susceptibility of the elderly to COVID‐19.

## 4 EXPERIMENTAL PROCEDURES

### 4.1 Mice

All the procedures involving mice were carried out in strict accordance with the recommendations in the Eighth Edition of the Guide for the Care and Use of Laboratory Animals of the National Research Council of the National Academies. All protocols were approved by Thomas Jefferson University's Institutional Animal Care and Use Committee. All young mice used in experiments were 6–12 weeks old and all aged mice were 18–20 months old. Both males and females were used. B6 mice were purchased from Charles river directly for experiments or as breeders. Aged B6 mice were obtained from the National Institute of Aging's aged mouse colony.

### 4.2 Viruses and infection

Ectromelia virus (ECTV)‐Moscow strain (ATCC VR‐1374), ECTV‐GFP, ECTV‐dsRED, and ECTVΔ166 were propagated in tissue culture as previously described (Xu et al., 2008). Mice were infected in the footpad with 3,000 plaque‐forming units (pfu) ECTV as indicated. For the determination of survival, mice were monitored daily and, to avoid unnecessary suffering, mice were euthanized and counted as dead when imminent death was certain as determined by lack of activity and unresponsiveness to touch. Euthanasia was according to the 2013 edition of the AVMA Guideline for the Euthanasia of Animals. For virus titers, the entire spleen or portions of the liver were homogenized in 2.5% FBS RPMI (Corning) using a Tissue Lyser (QIAGEN). Virus titers were determined on BSC‐1 cells as previously described (Xu et al., 2008).

### 4.3 Cell isolation

Mice were euthanized by cervical dislocation. Single‐cell suspensions were prepared from the footpad and skin‐draining popliteal lymph nodes. LNs were first incubated in Liberase TM (1.67 Wünsch units/ml) (Sigma) in PBS with 25 mM HEPES for 30 min at 37°C before adding PBS with 25 mM HEPES + 10% FBS to halt the digestion process, followed by mechanical disruption of the tissue with 21 gauge needles (BD) and 1 ml syringes (BD) and then filtered through a 70‐μm mesh. For skin, the footpads of mice were skinned and finely chopped up with surgical scissors in 2 ml safe‐lock tubes (Eppendorf). Processed skin was then incubated in Liberase TM (1.67 Wünsch units/ml) (Sigma) in PBS with 25 mM HEPES for 60 min at 37°C on a rotator before adding PBS with 25 mM HEPES + 10% FBS to halt the digestion process. Samples were then filtered through a 70‐μm filter.

### 4.4 Flow cytometry

To determine cellular responses in the LNs, intact LNs were incubated at 37°C for 1 hr in media containing 10 µg/ml brefeldin A and then made into single cell suspensions. The cells were then stained for cell surface molecules, fixed, permeabilized, and stained for intracellular molecules using the Cytofix/Cytoperm kit (BD) according to the manufacturer's instructions. The following Abs were used: BV786‐CD3 (Clone 17A2; Biolegend), BV605‐CD4 (Clone RM4‐5; Biolegend), BV785‐CD8 (Clone 53–5.8; Biolegend), APC/Fire^TM^750/ BV605/ BUV395‐CD11b (Clone M1/70; Biolegend), PE‐Cy7‐CD11c (Clone N418; Biolegend), BUV395‐CD19 (Clone 1D3; eBiosciences), AF647‐CXCL9 (Clone MIG‐2F5.5; Biolegend), Fitc‐CD45 (Clone 30‐F11; Biologend), Apc‐Cy7/ PerCP‐Cy5.5‐CD103 (Clone 2E7; Biolegend), APC/PE‐CD207 (4C7; Biolegend), Pacific Blue‐GR‐1 (RB6‐8C5; Biolegend), PE/Cy7/BV786‐IFNγ (XMG1.2; Biolegend), Pacific Blue/PerCP‐Cy5.5‐MHC II (I^ab^) (Clone AF6‐120.1; Biolegend), PerCP‐MULT1 (Clone 237104: R&D Systems), APC/BV605‐NK1.1 (PK136; Biolegend), BV785‐TCRβ (Clone H57‐597; BD), and For analysis, samples were acquired using a BD Fortessa flow cytometer (BD Biosciences), and data were analyzed with FlowJo software (TreeStar).

### 4.5 IFN‐γ neutralization

Experimental mice were injected with 100µg of α‐mouse IFN‐γ (Clone H22; BioXcell) intraperitoneally 24 hr prior to infection with WT ECTV.

### 4.6 RNA preparation and RT‐qPCR

Total RNA from LNs was obtained with the RNeasy Mini Kit (QIAGEN) as previously described (Rubio et al., 2013; Xu et al., 2015). First‐strand cDNA was synthesized with High Capacity cDNA Reverse Transcription Kit (Life Technologies). For *Gapdh, Evm003, Ccl2, Ccl7, Ifng, Cxcl9, Ifna4, Ifnb1,* and *Ifna‐non4* RT‐qPCR was performed using iTaq Universal SYBR Green with the following primers: *Gapdh*: tgtccgtcgtggatctgac and cctgcttcaccaccttcttg, *Evm003*: tctgtcctttaacagcatagatgtaga and tgttaactcggaagttgatatggta, *Ccl2:* catccacggttggctca *and* gatcatcttgctggtgaatgagt, *Ccl7:* ttgacatagcagcatgtggat and ttctgtgcctgctgctcata, *Ifng:* ttcaagacttcaaagagtctgaggta and gcaaaaggatggtgacatga, *Cxcl9:* cttttcctcttgggcatcat and gcatcgtgcattccttatca, *Ifna4:* gtcttttgatgtgaagaggttcaa and tcaagccatccttgtgctaa, *Ifnb1:* cacagccctctccatcaacta and catttccgaatgttcgtcct, *Ifna‐non4*: aagctgtgtgatgcaacaggt and ggaacacagtgatcctgtgg. For genes of interest, RNA (ECTV) from the contralateral lymph node (ndLN) of young mice infected with WT ECTV or ECTV‐Δ166 were used as controls to determine the fold change. In samples where no amplification was observed, CT values were adjusted to 40 for quantification purposes.

### 4.7 Statistical analysis

Statistical analysis was performed with Prism software (GraphPad Software Inc.) software. For survival studies, *p*‐values were obtained with the log‐rank (Mantel‐Cox) test. For all other studies, *p*‐values were determined using Mann–Whitney test, and when multiple groups had to be compared, the one‐way ANOVA with Tukey correction for multiple comparisons. For statistical significance, * = *p* < .05; ** = *p* < .0; *** = *p* < .001; **** = *p* < .0001.

## CONFLICT OF INTERESTS

The authors declare no conflict of interests.

## AUTHORS' CONTRIBUTIONS

Luis J. Sigal contributed to the conceptualization, analysis, administration, and supervision of the project, and the editing of the manuscript. Coby Stotesbury performed most of the experiments, analyzed results, and wrote the first draft of the manuscript. Eric B. Wong, Lingjuan Tang, Brian Montoya, Cory J. Knudson, and Carolina Melo‐Silva contributed with some experiments and/or preparation of essential reagents and analysis of results.

## Data Availability

This manuscript does not include large datasets.
